# *Amigo2*-upregulation in Tumour Cells Facilitates Their Attachment to Liver Endothelial Cells Resulting in Liver Metastases

**DOI:** 10.1038/srep43567

**Published:** 2017-03-08

**Authors:** Yusuke Kanda, Mitsuhiko Osaki, Kunishige Onuma, Ayana Sonoda, Masanobu Kobayashi, Junichi Hamada, Garth L. Nicolson, Takahiro Ochiya, Futoshi Okada

**Affiliations:** 1 Division of Pathological Biochemistry, Tottori University Faculty of Medicine, Yonago, Japan; 2 Chromosome Engineering Research Center, Tottori University, Yonago, Japan; 3 Health Sciences University of Hokkaido, School of Nursing and Social Services, Ishikari-Tobetsu, Japan; 4 Department of Molecular Pathology, The Institute for Molecular Medicine, South Laguna Beach, CA, USA; 5 Division of Molecular and Cellular Medicine, National Cancer Center Research Institute, Tsukiji, Chuo-ku, Tokyo, Japan

## Abstract

Since liver metastasis is the main cause of death in cancer patients, we attempted to identify the driver gene involved. QRsP-11 fibrosarcoma cells were injected into the spleens of syngeneic mice to isolate tumour sub-populations that colonize the liver. Cells from liver metastatic nodules were established and subsequently injected intrasplenically for selection. After 12 cycles, the cell subline LV12 was obtained. Intravenous injection of LV12 cells produced more liver metastases than QRsP-11 cells, whereas the incidence of lung metastases was similar to that of QRsP-11 cells. LV12 cells adhered to liver-derived but not to lung-derived endothelial cells. DNA chip analysis showed that *amphoterin-induced gene and open reading frame 2 (Amigo2*) was overexpressed in LV12 cells. siRNA-mediated knockdown of *Amigo2* expression in LV12 cells attenuated liver endothelial cell adhesion. *Ex vivo* imaging showed that suppression of *Amigo2* in luciferase-expressing LV12 cells reduced attachment/metastasis to liver to the same level as that observed with QRsP-11 cells. Forced expression of *Amigo2* in QRsP-11 cells increased liver endothelial cell adhesion and liver metastasis. Additionally, *Amigo2* expression in human cancers was higher in liver metastatic lesions than in primary lesions. Thus, *Amigo2* regulated tumour cell adhesion to liver endothelial cells and formation of liver metastases.

The pathogenesis of metastasis has been investigated for more than 150 years, and metastasis remains the cause of over 90% of cancer deaths^1^,^2^. Among the common sites of distant metastases, liver is the most frequent site (59%)^3^. Hence, there is an urgent need to identify the molecule(s) that facilitate liver metastasis in order to develop potential preventive and therapeutic target(s) for liver metastases.

Metastases are thought to originate from cell sub-populations within a biologically heterogeneous primary tumour^4^,^5^. Experimental and clinical studies indicate that the metastatic process is highly selective and that metastases can be clonal in origin^6^,^7^ and are not the result of adaptation of tumour cells to a secondary site^8^. Whole-genome sequencing has also revealed profound differences in gene expression between local and disseminated tumours^9^, suggesting that information regarding primary tumours alone is insufficient to determine optimal therapeutic strategies. Therefore, an understanding of the molecular differences among phenotypes of metastasis-initiating tumour cells in the primary growing tumour is needed^10^.

An *in vivo* selection procedure can be used to obtain cell sublines with increased liver metastatic potential, and this method can provide a powerful tool to study those intrinsic properties that distinguish metastatic from non-metastatic cells^11^. For example, tumour cells can be injected intrasplenically into mice resulting in the formation of liver metastases. Tumour cells from the liver-metastatic lesions can be isolated and established in culture. After multiple rounds of selection for liver colonization, the *in vivo*-selected variants have higher liver-metastatic potential than the parental tumour cell line^12^. This procedure has been applied to human tumour cells of the colon, pancreas and stomach^13^,^14^,^15^. However, to establish a liver metastasis model using human tumour cell lines, immunodeficient mice must be used as the host^16^. Recent evidence indicates that the tumour microenvironment, which is composed of a variety of cell types, including activated fibroblasts, inflammatory cells and vascular endothelial cells, is selective and required for acquisition of the metastatic phenotype^16^,^17^,^18^. For example, T helper 2 cells and regulatory T cells in the tumour microenvironment may be involved in mammary cancer metastasis^19^,^20^. In addition, species specificity may be important for some cytokine-signalling pathways, for example, in interactions between the IFNγ/IFNγ receptor and the IL-6/IL-6 receptor^21^,^22^. In immunodeficient animal hosts the natural selection process and the metastatic cascade are slightly different than those in humans, and thus the selection of human metastatic variants in animals may yield molecular differences that are dissimilar compared to metastatic tumours obtained from human cancer patients^23^. On the other hand, syngeneic models reflect the natural pathogenesis of carcinogenesis and tumour progression processes^23^. We therefore chose a syngeneic mouse model to obtain liver metastatic cell sublines in order to examine differences related to the metastatic phenotype.

In the present study, sequential *in vivo* selection (twelve cycles) of QRsP-11 mouse fibrosarcoma-derived cells was used to establish the LV12 cell subline, which has markedly enhanced liver-metastatic potential compared to the parental cells. We explored the differential expression of liver metastasis-responsible gene(s) in the parental and the selected metastatic subline by comparing their gene expression profiles. *Amphoterin-induced gene and open reading frame 2 (Amigo*2) was identified as an overexpressed gene in LV12 cells, and knockdown of *Amigo2* expression resulted in suppression of liver metastasis via attenuation of tumour cell adhesion to liver endothelial cells. Conversely, forced expression of *Amigo2* in the parental cells induced increased liver endothelial cell adhesion and liver metastasis. We also confirmed that *Amigo2* expression regulates liver metastasis in human cancers. These results, for the first time, indicate that *Amigo2* plays a key role in liver metastasis formation.

## Results

### *In vivo* selection of liver-metastasizing sublines from QRsP-11 cells

**Figure 1:**
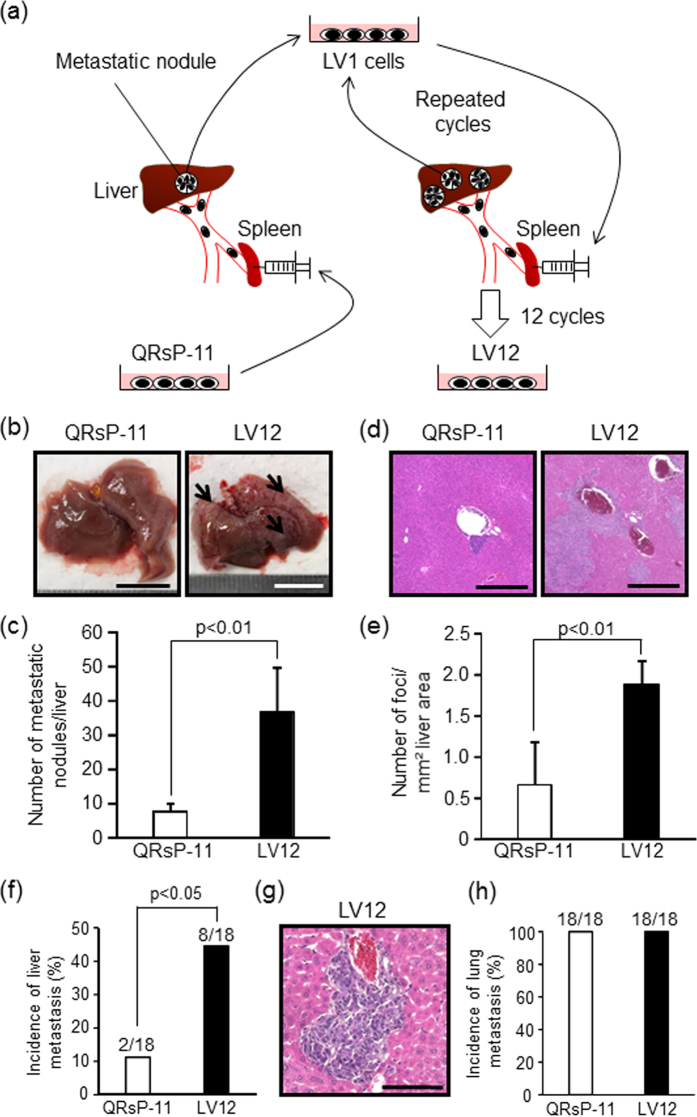
LV12 cells possess a high liver-metastatic potential and give rise to multiple liver colonies by intrasplenic and intravenous injections. (**a**) Schematic representation of *in vivo* sequential selection of a liver-metastatic variant subline (LV12) from QRsP-11 mouse fibrosarcoma cells. Metastatic nodules in the livers of C57BL/6 mice, which formed from 1 × 10^6^ QRsP-11 cells previously injected into the spleen, were harvested and a cell culture subline was established. These cells were then injected into the spleens of other mice, and liver metastases subsequently formed. The metastatic foci were excised and expanded as a new subline. This procedure was repeated 12 times, yielding a cell subline designated as LV12. (**b**) Macroscopic views of liver metastasis formation 7 days after intrasplenic injection of 1 × 10^6^ LV12 or QRsP-11 cells. Arrows indicate representative metastatic nodules. Scale bar: 10 mm. (**c**) The numbers of metastases on the liver surface were determined. Bar graphs show means ± SD (n = 5 in each group). (**d**) Representative H&E staining of the livers from Fig. 1b. Scale bar: 500 μm. (**e**)The number of metastatic foci per mm^2^ of liver area was determined using image analysis software. Bar graphs show means ± SD (n = 5 in each group). (**f**) The incidence of liver metastatic colonization 7 days after tail vein injection of 1 × 10^6^ LV12 or QRsP-11 cells was evaluated using histology. Bar graphs show means ± SD from three independent experiments with similar results (n = 18 in each group). (**g**) Microscopic appearance of the liver metastatic foci formed by LV12 cells. Scale bar: 100 μm. (**h**) The incidence of lung metastasis was determined by the presence of metastatic nodules on the lung surface on day 7 post-injection. The incidence of metastatic colonization is indicated above each bar (number of mice with metastasis/number of mice examined).

To isolate sublines of QRsP-11 fibrosarcoma cells with high liver-metastatic properties, the QRsP-11 cells were subjected to an *in vivo* selection protocol that involved repeated, sequential intrasplenic injections (Fig. 1a). Liver metastatic colonies were excised aseptically and expanded as *in vitro* cultured cell sublines. The established cell sublines were injected intrasplenically and these procedures were then repeated. The liver/body weight ratio increased, and the survival period was shortened following each successive selection cycle (Supplementary Table S1). After 12 rounds of *in vivo* selection, a highly metastatic variant LV12 cell subline was obtained that induced significant changes in these parameters versus the parental cells. We therefore utilized these LV12 cells for further investigation. The LV12 cell phenotype of liver metastasis was stable, since the cells still formed liver metastasis after maintenance for at least 6 months under culture conditions (data not shown).

### Increase in the liver metastatic potential of LV12 cells after intrasplenic injection

To confirm the higher liver-metastatic potential of LV12 cells compared to QRsP-11 cells, mice were examined on day 7 after intrasplenic injection. The average number of metastatic nodules on the liver surfaces of mice injected with LV12 cells was significantly higher (36.6 ± 13.2) than that found in mice injected with QRsP-11 cells (7.4 ± 2.5, *p* < 0.01, Fig. 1b,c). Numerous metastases were also found in liver parenchyma after LV12 cell injection, whereas only a few foci were formed in the periphery of the portal vein after QRsP-11 cell injection (Fig. 1d). As shown in Fig. 1e, the number of metastatic foci per mm^2^ of liver surface area was also significantly higher in LV12 cell-injected mice (1.8 ± 0.4) than in QRsP-11 cell-injected mice (0.7 ± 0.5, *p* < 0.01).

### LV12 cells colonize the liver after intravenous injection

To determine whether LV12 cells possessed preferential liver metastasis, LV12 cells or QRsP-11 cells were injected into tail veins, a route that allows for initial passage of tumour cells through the lungs. Seven days after intravenous injection of LV12 cells the incidence of liver metastasis (8 out of 18; 44.4%) was higher than found with injected QRsP-11 cells (2 out of 18; 11.1%, *p* < 0.05, Fig. 1f,g). However, there was no difference in the incidence of lung metastasis between the two cell lines (Fig. 1h), suggesting that LV12 cells have a higher capacity to colonize the liver.

### LV12 cells preferentially adhere to liver endothelial cells

**Figure 2:**
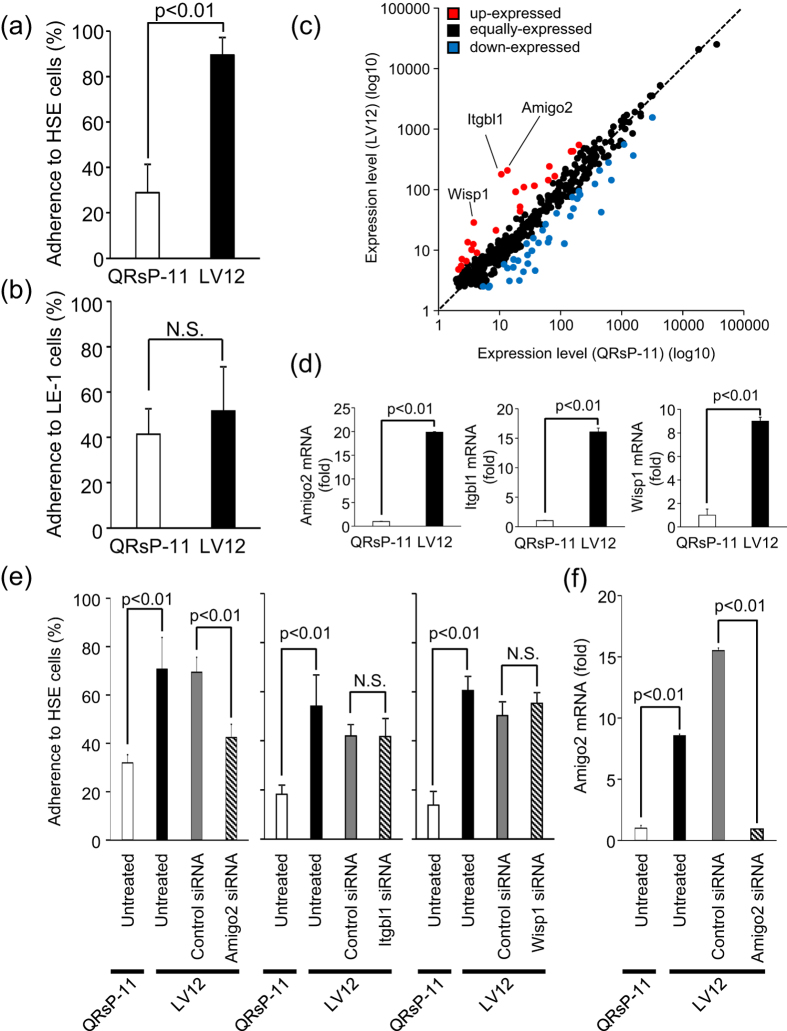
Amigo2 functions in the adhesion of LV12 cells to liver endothelial cells. (**a**,**b**) LV12 and QRsP-11 cells were labelled with fluorescent dye and were placed onto endothelial cells derived from liver (HSE) or lung (LE-1) for 30 min. The percentages of adherent cells were determined by measuring fluorescence intensity. Bar graphs show means ± SD (n = 5 in each group). (**c**) Scatter plot of adhesion-related mRNA expression profiles of LV12 cells and QRsP-11 cells. *Amigo2, Itgbl1* and *Wisp1* were included in the upregulated genes in LV12 cells. (**d**)The relative levels of *Amigo2, Itgbl1* and *Wisp1* mRNA in LV12 cells compared to QRsP-11 cells as measured using qRT-PCR. Bar graphs show means ± SD (n = 5 in each group). (**e**) LV12 cells were transfected with siRNA targeting *Amigo2, Itgbl1, Wisp1* or with control siRNA. After 24 h, the cells were harvested and their adhesion to HSE cells was assayed. Bar graphs show means ± SD (n = 5 in each group). (**f**) Downregulation of *Amigo2* mRNA expression by *Amigo2* siRNA. Transfection of LV12 cells was confirmed using qRT-PCR. Bar graphs show means ± SD (n = 4 in each group).

To understand the mechanism of increased liver metastatic potential of LV12 cells we focused on adhesion of tumour cells to liver endothelial cells, since this is an initial and critical step in the formation of blood-borne metastases. Cell adhesiveness was evaluated by using our previously established *in vitro* assay^24^. As shown in Fig. 2a, the adhesion of LV12 cells to the liver endothelial (HSE) cells (89.5 ± 7.6%) was significantly higher than that of QRsP-11 cells (28.8 ± 12.6%: *p* < 0.01), whereas adhesion to lung endothelial (LE-1) cells was similar in both cell lines (Fig. 2b). These results suggested that metastasis of LV12 cells to the liver might be due to the adhesiveness of LV12 cells to liver endothelial cells, which was markedly enhanced compared to that of QRsP-11 cells.

### Identification of adhesion-related molecules overexpressed in LV12 cells

To identify adhesion-related molecules that were overexpressed in LV12 cells DNA chip analysis (17,448 genes) was performed (Fig. 2c). These microarray data have been deposited in the Gene Expression Omnibus database (GEO; http://www.ncbi.nlm.nih.gov/geo/). Genes with functional annotations related to adhesion (428 genes) were chosen as suggested by Gene Ontology. Taking a two-fold change as a cut-off level, 23 genes were upregulated and 38 genes were downregulated in LV12 cells versus the parental cells (Supplementary Table S2). We confirmed using qRT-PCR analysis that the mRNAs of three of the highly expressed genes, *amphoterin-induced gene and open reading frame 2 (Amigo2*), *Integrin beta-like 1 (Itgbl1*) and *Wnt-1-inducible signalling pathway protein 1 (Wisp1*), were upregulated in LV12 cells compared to the parental line (Fig. 2d). We found that significant and consistent upregulation of *Amigo2* could be detected in the LV12 cell line that was established from QRsP-11 cells after at least 4 cycles of *in vivo* repeated selection (Supplementary Table S1).

### *Amigo2* mediates LV12 cell adhesion to liver endothelial cells

Adhesion of LV12 cells to liver endothelial HSE cells was inhibited by prior transfection of *Amigo2* siRNA into LV12 cells (*p* < 0.01), but not by transfection of *Itgbl1* or *Wisp1* siRNA (Fig. 2e). We confirmed that *Amigo2* (Fig. 2f), *Itgbl1* and *Wisp1* (data not shown) mRNA levels were suppressed by the relevant siRNA transfections.

Since the Amigo family consists of three molecules (Amigo1, Amigo2 and Amigo3) that associate through homophilic or heterophilic binding^25^, we compared the mRNA expression of each of these molecules in LV12 cells with that in QRsP-11 cells. *Amigo2* mRNA was significantly upregulated in LV12 cells compared to QRsP-11 cells (Supplementary Fig. S1). Moreover, we found that all three Amigo molecules were expressed on liver endothelial HSE cells, whereas none of these Amigo molecules were expressed on lung endothelial LE-1 cells (Supplementary Fig. S2). Furthermore, adhesion of LV12 cells to LE-1 cells was not suppressed by prior transfection of *Amigo2* siRNA into LV12 cells (Supplementary Fig. S3). Therefore, the preferential and selective adhesion of LV12 cells to HSE cells is mediated by Amigo molecules on both of these cell types.

Regarding the observed lung metastasis, we speculated that it will involve cell adhesion molecule(s) other than *Amigo2*, since there was no difference in the frequency of lung metastatic incidence (Fig. 1h) or adherence to lung endothelial cells (Fig. 2b) between LV12 cells and QRsP-11 cells which display different *Amigo2* expression (Fig. 2d).

### *Amigo2* is involved in the liver metastasis of LV12 cells *in vivo*

**Figure 3:**
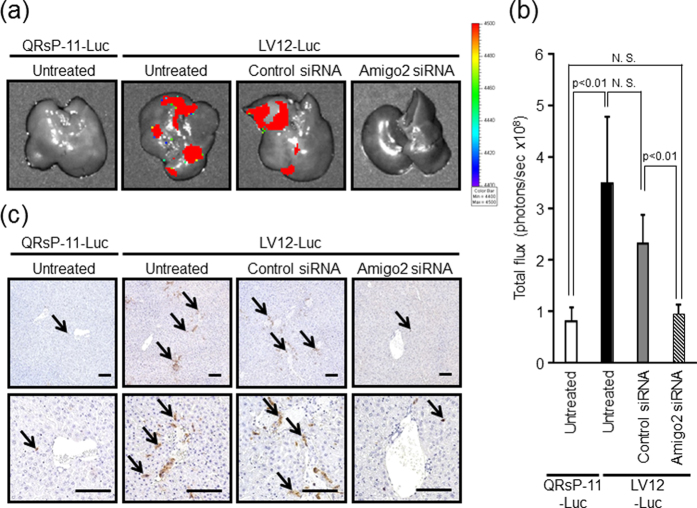
RNA interference-mediated Amigo2 suppression reduces intrahepatic attachment of LV12 cells. (**a**) An expression vector for *firefly luciferase* was introduced into LV12 and QRsP-11 cells (LV12-Luc and QRsP-11-Luc, respectively). Representative bioluminescent images of livers 4 hours after intrasplenic injection of 5 × 10^6^ QRsP-11-Luc or LV12-Luc cells transfected with either *Amigo2* or control siRNA are shown. (**b**) Quantification of total flux (photons per sec) from the livers shown in Fig. 3a. Bar graphs show means ± SD (n = 4 in each group). (**c**) Tumour cells in the liver tissues were detected using immunohistochemistry with an anti-luciferase antibody. LV12-Luc or QRsP-11-Luc cells are indicated by arrows. Top, ×10 low-power field; bottom, ×20 high-power field. Scale bar: 100 μm.

To investigate whether *Amigo2* is required for an early phase of liver metastasis (tumour cell attachment to liver endothelial cells), we transfected a *firefly luciferase* expression vector into LV12 cells (LV12-Luc cells) and QRsP-11 cells (QRsP-11-Luc cells). Four hours after intrasplenic injection of these luciferase expression transfectants, the degree of tumour cell attachment to the liver was evaluated using quantitative bioluminescence imaging. LV12-Luc cells but not QRsP-11-Luc cells could be detected in the liver. *Amigo2* knockdown by its siRNA led to a reduction in the attachment of LV12-Luc cells to the level of that observed in untreated QRsP-11-Luc cells (Fig. 3a,b). In addition, control siRNA transfections did not alter liver adhesion of LV12-Luc cells compared to untreated cells (Fig. 3a,b).

As shown in Fig. 3c, both untreated and control siRNA-treated LV12-Luc cells were observed in the liver, whereas LV12-Luc cells in which *Amigo2* was downregulated, or untreated QRsP-11-Luc cells, were rarely observed in the liver parenchyma. These data demonstrate that *Amigo2* expression in LV12 cells is responsible, at least in part, for intrahepatic attachment *in vivo*. We also confirmed that transfection of the *Luc* gene construct did not alter adhesiveness to liver endothelial cells (Supplementary Fig. S4).

**Figure 4:**
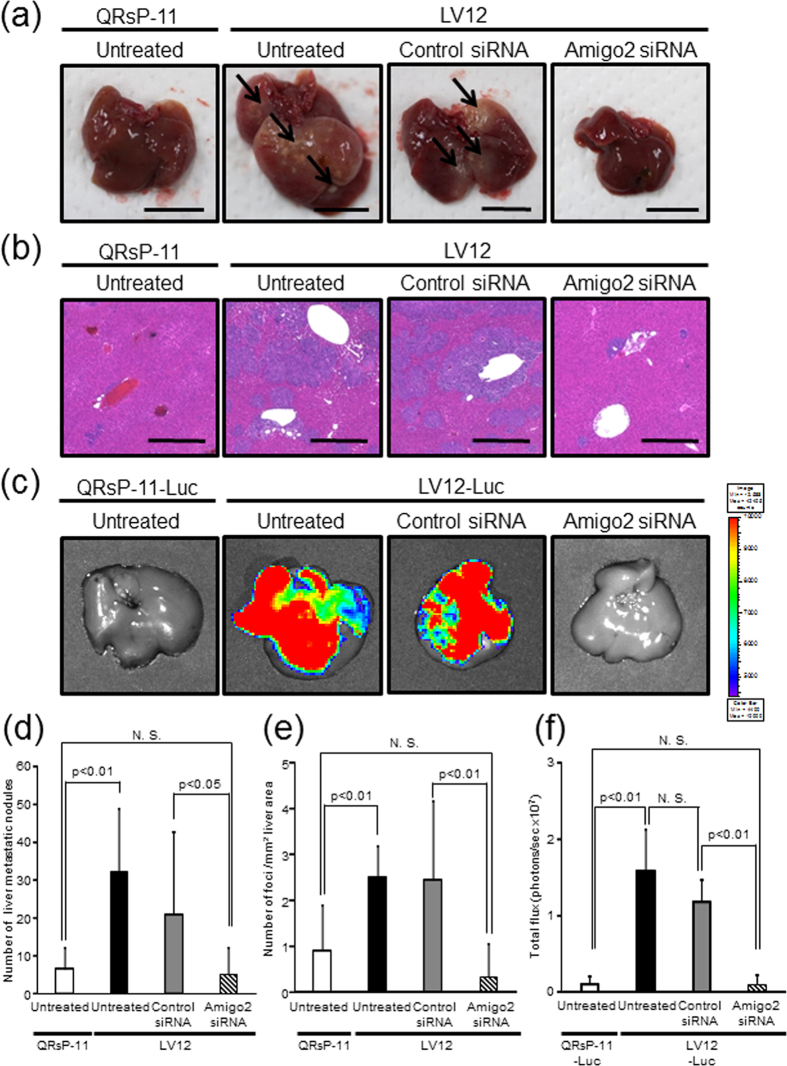
Downregulation of Amigo2 mRNA in LV12 cells decreased liver metastasis. (**a**) Representative pictures show the macroscopic metastatic nodules (indicated by arrows) on the liver surface 7 days after the mice were injected intrasplenically with LV12 cells transfected with *Amigo2* or control siRNA. Scale bar: 10 mm. (**b**) H&E staining of the liver metastatic foci. Scale bar: 500 μm. (**c**) Representative bioluminescent images of livers 7 days after intrasplenic injection of QRsP-11-Luc or LV12-Luc cells transfected with either *Amigo2* or control siRNA are shown. (**d**) Metastatic nodules on the liver surface in individual mice were counted. Bar graphs show means ± SD from two independent experiments with similar results. QRsP-11 and untreated LV12 cells, n = 12; control siRNA or *Amigo2* siRNA-transfected LV12 cells, n = 11. (**e**) The number of metastatic foci per mm^2^ of liver area was determined. Bar graphs show means ± SD from two independent experiments with similar results. QRsP-11 and untreated LV12 cells, n = 12; control siRNA or *Amigo2* siRNA-transfected LV12 cells, n = 11. (**f**) Quantification of total flux (photons per sec) from the livers shown in Fig. 4c. Bar graphs show means ± SD (n = 9 in each group).

To clarify the possible role of *Amigo2* in the formation of liver metastases, LV12 cells transfected with or without *Amigo2* siRNA were injected into the spleens of mice, and the mice were then sacrificed on day 7. LV12 cells transfected with or without control siRNA formed macroscopic liver metastasis, whereas *Amigo2*-siRNA knockdown-LV12 cells dramatically lost their liver-metastatic potential, which was reduced to the level found with untreated QRsP-11 cells (Fig. 4a,d). We also showed that the number of microscopic metastatic foci was decreased by inhibition of *Amigo2* expression in LV12 cells compared to control siRNA-transfected cells (Fig. 4b,e). We next quantified *Amigo2*-mediated liver metastasis using a bioluminescence imaging system. *Amigo2* knockdown by its siRNA led to a significant reduction in liver metastasis of LV12-Luc cells, which was reduced to the total photon flux level of that observed for untreated QRsP-11-Luc cells (Fig. 4c,f). In addition, control siRNA transfected LV12-Luc cells did not show altered metastatic potential compared to untreated cells (Fig. 4c,f). These data indicated that increased *Amigo2* expression in LV12 cells is necessary for the establishment of liver metastases.

### Increased expression of *Amigo2* is sufficient for acquisition of liver endothelial cell adhesion and liver metastasis formation

**Figure 5:**
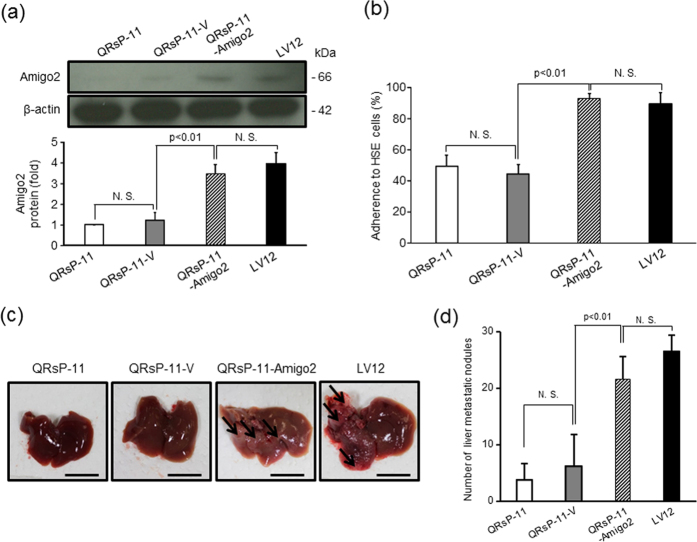
Amigo2 overexpression in QRsP-11 cells promotes their adhesion to liver endothelial (HSE) cells and liver metastasis. (**a**) QRsP-11 cells were stably transfected with an empty (QRsP-11-V) or an *Amigo2* expression (QRsP-11-Amigo2) vector. Amigo2 overexpression was confirmed by western blot analysis. β-actin served as the internal control. Bar graphs show means ± SD (n = 3 in each group). Full-length blots were presented in Supplementary Fig. S5. (**b**)The adhesion of the transfectants to HSE liver endothelial cells was quantified. Bar graphs show means ± SD (n = 5 in each group). (**c**) Macroscopic observation of the livers of mice 7 days after intrasplenic injection of control or *Amigo2*-overexpressing QRsP-11 cells are shown. The arrows highlight metastatic nodules. Scale bar: 10 mm. (**d**) The liver surface nodules in each mouse were counted. Bar graphs show means ± SD (n = 5 in each group).

To verify the role of *Amigo2* in the acquisition of liver metastatic properties, we introduced the pEZ-M02-*Amigo2* expression vector into the parental QRsP-11 cells (QRsP-11-Amigo2). Western blot analysis demonstrated that Amigo2 protein expression in the QRsP-11-Amigo2 cells, but not in empty vector-transfected QRsP-11 cells (QRsP-11-V), was increased compared to the parental QRsP-11 cells (Fig. 5a). This increased Amigo2 expression reached almost the same level as that observed in untransfected LV12 cells. Full-length blots were presented in Supplementary Fig. S5.

We then analyzed the attachment of these transfectants to liver endothelial HSE cells. QRsP-11-Amigo2 cells, but not QRsP-11-V cells, showed increased attachment to HSE cells compared to parental cells, and this attachment reached the same level as that observed in LV12 cells (*p* < 0.01) (Fig. 5b).

To determine if expression of *Amigo2* in QRsP-11 cells is sufficient for acquisition of liver metastasis, we intrasplenically injected these transfectants. After 7 days, QRsP-11-Amigo2 cells developed metastatic foci to the same level as that observed in untreated LV12 cells (Fig. 5c,d). Additionally, QRsP-11-V cells did not promote liver metastatic ability compared to untreated QRsP-11 cells.

### Expression of Amigo2 is elevated in liver metastatic lesions in human cancers

**Figure 6:**
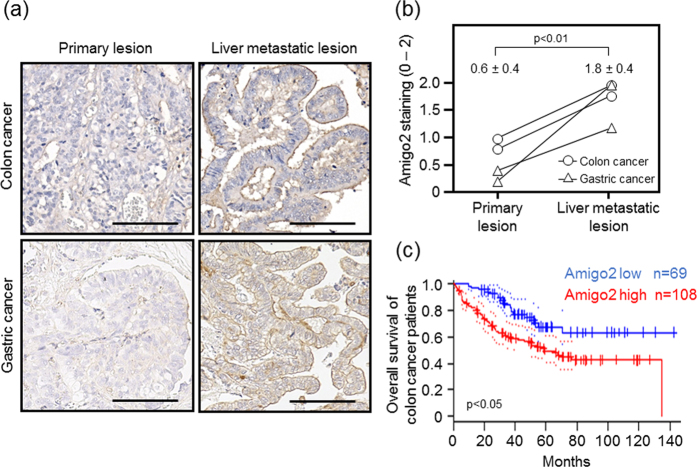
Amigo2 is overexpressed in liver metastases in human colon and gastric carcinomas. (**a**) Immunohistochemical staining of Amigo2 in matched primary and liver metastatic lesions of human colon and gastric cancers. Scale bar: 100 μm. (**b**) Amigo2 staining intensity in paired specimens of primary colon and gastric carcinomas and their metastases. Results are shown as means ± SD (n = 4 in each group). (**c**) Kaplan-Meier analysis of overall survival in colon cancer patients with high *versus* low *Amigo2* mRNA expression. Dotted lines indicate 95% confidence intervals for each group. These data were obtained from PrognoScan (http://www.abren.net/PrognoScan/).

To determine if it was possible to extrapolate the results of the animal experiments to human cancers, we next analysed Amigo2 expression in clinically matched primary and liver metastatic colon and gastric cancers using immunohistochemistry. Faint staining of Amigo2 was detected in primary tumour lesions (Fig. 6a). However, in all of the cases examined, increased Amigo2 expression was observed in the matched liver metastatic foci, especially on the tumour cell surface (Fig. 6a). The Amigo2 staining intensity scores were an average of 1.8 ± 0.4 in the liver metastatic lesions, which were significantly higher than the scores in the primary tumour lesions (0.6 ± 0.4, *p* < 0.01, Fig. 6b). Furthermore, PrognoScan-based Kaplan-Meier survival analysis revealed a correlation between higher *Amigo2* expression levels and shorter survival times in 177 colon cancer patients (*p* < 0.05, Fig. 6c).

## Discussion

In this study, we established the LV12 subline from QRsP-11 fibrosarcoma cells by sequential *in vivo* selection of metastatic liver foci that were formed following intrasplenic injection. By using these liver-metastatic cells, we found for the first time that the *Amigo2* gene is likely to play a central role in fibrosarcoma cell adhesion to liver endothelial cells as well as in the formation of metastatic foci in the liver.

Seven cell surface molecules (sialyl Lewis A, sialyl Lewis X, GM1b, GD1α, integrin α4β1, annexin A2 and A6) have been previously proposed to mediate tumour cell adhesion to liver endothelial cells^26^,^27^,^28^,^29^,^30^,^31^. However, a major limitation in confirming the importance of those molecules is that they were not tested for their involvement in liver metastasis formation *in vivo*^26^,^27^,^28^,^29^,^30^,^31^. Our study is the first to show the involvement of a specific driver gene product in *in vivo* liver metastasis formation as well as in clinical tumour samples.

Amigo2 is expressed in various organs, such as the cerebrum, cerebellum, retina, lung, liver, kidney, small intestine, spleen and testis^25^. Amigo2 has been found to play a pathological role in tumour growth as well as in cell adhesion to, and migration through collagen of gastric cancer cells^32^. We showed here that Amigo2 mediates tumour cell-liver endothelial cell adhesion, intrahepatic cell attachment and in establishment of liver metastasis.

Structural analysis has shown that the extracellular region of Amigo family proteins has leucine-rich repeat (LRR) domains^33^. Among proteins that contain the LRR domain, such as increased expression of leucine-rich repeat-containing G protein-coupled receptor 5, toll like receptor 4 and tropomyosin receptor kinase B, all have been associated with liver metastasis of colon or pancreatic cancers^34^,^35^,^36^. Thus, Amigo2 is the fourth liver metastasis regulatory LRR-containing protein identified. LRR domains of a cell surface protein mediate cell-cell adhesion^33^, suggesting that Amigo2 is likely to mediate adhesion to liver endothelial cells and to promote liver metastasis via its LRR domains. Future investigations focused on LRR domain-mutated Amigo2 are needed to determine whether the LRR domain plays an important role in cell adhesion and liver metastasis.

Previous experimental studies have identified 13 liver metastasis-associated genes by use of sequential intrasplenic selection methods in immunodeficient mice to obtain liver-metastatic variants of human cancer cells and compare these variants to parental cancer cells^37^,^38^,^39^,^40^,^41^,^42^,^43^. However, in only one out of these 13 molecules (8%) has been verified to be expressed in clinical specimens and to be associated with liver metastasis^43^. Thus, identification of metastasis-associated genes and molecules in xenograft models may not be useful for predicting genes/gene expression that are clinically relevant. In contrast, integrin α2 44 and the Amigo2 protein (the present study) have been identified as molecules responsible, in part, for liver metastasis in a syngeneic animal model using mouse tumour cells and C57BL/6 mice. Interestingly, elevated expression of both genes was also confirmed in metastatic lesions of human specimens. Therefore, to identify driver gene(s) and molecules(s) that are potentially relevant in a clinical setting it may be better to use a syngeneic model as was done in our study rather than a xenogeneic model. The use of a syngeneic model may increase the probability of identifying clinically relevant molecules that may function as functional markers of metastasis or therapeutic targets.

We noticed that host cells that co-existed with tumour cells facilitated the malignancy of the tumour cells^45^. Inflammatory cells are typical of such host cells. To avoid the co-existence of such cells, we eliminated host cell contamination during establishment of the tumour cell line from the metastatic nodules by repeated passages of the cells in culture^46^. Moreover, tumour-to-tumour cell and/or tumour-to-host cell fusion is another possible mechanism of gaining malignancy^47^. In particular, in terms of acquisition of a metastatic phenotype, fusion between normal motile cells and tumour cells could underlie metastasis^48^. Since such cell fusion-derived progeny cells have a proclivity for polyploidy^47^, we examined the mean number of chromosomes in the cell lines in our study. The mean number of chromosomes in LV12 and parental QRsP-11 cells was 67 and 70, respectively (Data not shown). These observations suggested that contamination of LV12 cells with normal cells or LV12 cell fusion is not a likely explanation of the acquisition of a liver metastatic phenotype by the LV12 cells.

We assumed two possible mechanisms/hypotheses to explain the Amigo2-mediated cell adhesion. Firstly, we considered that Amigo2 itself functions as a cell-to-cell adhesion molecule. The selective binding of Amigo2-expressing LV12 cells to hepatic but not to pulmonary endothelial cells is likely to be mediated through homophilic or heterophilic binding among the three homologous proteins that comprise the Amigo family, i.e., Amigo1, Amigo2, and Amigo3 25. We showed that all of these Amigo family molecules are expressed on HSE liver endothelial cells; however, none of these Amigo molecules are expressed on LE-1 lung endothelial cells (Supplementary Fig. S2). Additionally, only *Amigo2* showed significantly higher expression in LV12 cells compared to parental QRsP-11 cells (Supplementary Fig. S1). Moreover, forced expression of *Amigo2* in QRsP-11 cells induced increase adherence to liver endothelium (Fig. 5b) and also increased metastasis to liver (Fig. 5c,d). We therefore concluded that the selective adhesion of LV12 cells to liver endothelial cells may be due to interactions between the Amigo family molecules that are expressed on both cell types. Our second hypothesis was that Amigo-2 may induce the expression/activation of integrins or adhesion-related kinases such as focal adhesion kinase (FAK) and Src. In our assessment of this possibility, we found that LV12 cells express 61 adhesion-related molecules (Supplementary Table S2). Of these molecules, the expression level of *integrin beta-like 1 (Itgbl1*) and *integrin beta 7 (Itgb7*) was increased in LV12 cells 16.5-fold and 4.3-fold, respectively, compared to that in QRsP-11 cells. As shown in Fig. 2e, siRNA-mediated downregulation of *Itgbl1* did not alter the adhesiveness of LV12 cells to liver endothelial cells. Moreover, we ascertained that downregulation of *Itgb7* in LV12 cells did not reduce their liver metastatic capacity (data not shown). Therefore, our investigations up to the present date suggest that it is unlikely that these integrins are involved in the adhesion observed in our system. We are currently undertaking a study to determine the precise mechanism(s) by which selective and firm adhesion can be brought about by Amigo2 expression.

We found that Amigo2 was more strongly expressed at the surfaces of cells in liver metastatic lesions compared to its expression in primary lesions of human colon and gastric cancers (Fig. 6a,b). These results may verify the involvement of upregulation of *Amigo2* gene expression in the formation of liver metastases in clinical samples. Increased expression of *Amigo2* also correlated with poor prognosis in colon cancer patients (Fig. 6c). A similar result was obtained by Kaplan-Meier Plotter analysis of gastric cancer patients (logrank *P* = 0.00024, http://kmplot.com/analysis/index.php? p=service&start=1). These findings suggest that Amigo2 is potentially useful as a prognostic marker in colon and gastric carcinomas.

In summary, we have shown that Amigo2, which is a member of the Amigo family that shows high species conservation, may be involved in determining liver metastasis by preferential adhesion of the tumour cells to liver endothelial cells. Targeting Amigo2 may create opportunities for the development of novel strategies to prevent liver metastasis establishment.

## Materials and Methods

### Animals

Female C57BL/6 mice (5 weeks-old) were obtained from Nippon SLC (Hamamatsu, Japan) and maintained under specific-pathogen-free conditions with light-dark cycles (12 h each) at 23 ± 3 °C and 50 ± 10% humidity at the Institute for Animal Experimentation of Tottori University. Animals were used after one week of acclimation. Diet and water were supplied and consumed *ad libitum* throughout the experiments. All experimental procedures and protocols were approved by the Institutional Animal Care and Use Committee of Tottori University (Permission No. 14-Y-14), and were carried out in accordance with their approved guidelines.

### Cell lines and culture conditions

The origin and characteristics of the QRsP-11 mouse fibrosarcoma cell line have been described previously^49^,^50^. QRsP-11 and its highly metastatic derivative subline and their transfectants were maintained in Eagle’s minimum essential medium (05900, Nissui Pharmaceuticals, Tokyo, Japan) containing 8% foetal bovine serum (FBS, 1370978, GIBCO, Gaithersburg, MD, USA). Mouse hepatic sinusoidal (HSE) and lung (LE-1) endothelial cells were maintained with a mixture of F12 medium and Dulbecco’s modified Eagle medium (05910 and 05919, respectively, Nissui Pharmaceuticals) supplemented with 10% FBS^51^. Cultures were maintained at 37 °C in an atmosphere of 95% air and 5% CO_2_.

### Establishment of a highly liver-metastatic cell subline

QRsP-11 cells (1 × 10^6^) were injected into the spleens of anaesthetised mice. The mice were sacrificed when they became moribund. Liver metastatic colonies were aseptically excised, mechanically disaggregated, and established as the LV1 cell subline in culture. The tumour cell line was passaged at least four times in culture to eliminate host cell contamination. The detailed procedures have been described elsewhere^46^. These cells were then injected intrasplenically in the same manner as the previous injections. This process was repeated until both increased liver-to-body weight ratios and a shortened survival period were significantly different to those observed following injection of the parental QRsP-11 cell line.

### Evaluation of the liver metastatic potential of LV12 cells injected into the spleen

Mice were injected intrasplenically with 1 × 10^6^ LV12, *Amigo2*-expressing QRsP-11 or empty vector-expressing QRsP-11 cells. Seven days later, the numbers of nodules on liver surfaces were counted. Images of these nodules were obtained using a digital microscope (BZ-X700, Keyence, Osaka, Japan) and were analysed with BZ-H3C software (Keyence) to determine the average number of metastatic foci *per* mm^2^ of liver surface area.

### Evaluation of liver and lung metastases of LV12 cells injected intravenously

LV12 or QRsP-11 cells (1 × 10^6^) were injected into the tail veins of mice. The incidence of liver and lung metastasis was evaluated histologically on day 7 post-injection.

### Tumour cell-endothelial cell adhesion assay

The tumour cell adhesion assay was performed according to a previously reported method^24^. Briefly, a 96-well plate (165305, Thermo Fisher Scientific, Waltham, MA, USA) was coated with 1% gelatine (074-02761, Wako, Osaka, Japan) for 16 h. A total of 8 × 10^3^ endothelial cells were seeded in each well after removing the gelatine solution. Tumour cells (2 × 10^5^) previously labelled with the PKH67 green fluorescent dye (PKH67GL-1KT, Sigma Aldrich, St. Louis, MO, USA) were then placed onto HSE or LE-1 endothelial cell monolayers in the wells and incubated for 30 min. The non-adherent cells were removed by washing with PBS, and the adherent cells were quantified with a fluorescent plate reader (Infinite M200 PRO, Tecan, Männedorf, Switzerland) at an excitation of 485 nm and an emission of 535 nm. The percentage of adherence was calculated as the fluorescence ratio (post-wash fluorescence/pre-wash fluorescence) × 100.

### RNA extraction and DNA microarray analysis

Total RNA was extracted from tumour cells using the miRNeasy Mini Kit (217004, Qiagen, Valencia, CA, USA) according to the manufacturer’s protocol. DNA microarray analysis of LV12 cells *versus* QRsP-11 cells was performed using the 3D-Gene™ DNA chip (Mouse Oligo chip 24 K, Toray, Tokyo, Japan).

Total RNA obtained from both cell types was labelled with Cy3 or Cy5 using the Amino Allyl MessageAMP II aRNA Amplification Kit (AM1753, Thermo Fisher Scientific). The Cy3- or Cy5-labelled aRNA pools were hybridized to the array for 16 h at 37 °C, using the supplier’s protocols (http://www.3d-gene.com). Signals were scanned using a 3D-Gene Scanner 3000 (Toray). Detected signals for each gene were normalized by a global normalization method (Cy3/Cy5 ratio median = 1). A total of 428 genes was selected based on Gene Ontology annotation terms. Genes with Cy3/Cy5 normalized ratios greater than 2.0 or less than 0.5 were defined, respectively, as commonly up- or downregulated genes. The microarray data have been deposited in the Gene Expression Omnibus database (GEO; http://www.ncbi.nlm.nih.gov/geo/) under the accession number GSE88923.

### cDNA preparation and qRT-PCR analysis

For qRT-PCR, 500 ng of total RNA was used for cDNA synthesis in a 10 μl reaction mixture containing PrimeScript^TM^ RT Master Mix (RR036A, Takara Bio, Shiga, Japan). PCR amplification of cDNA was performed by using SYBR Premix Ex Taq II (RR820, Takara Bio). The primer sequences are shown in Supplementary Table S3. The PCR cycles consisted of 5 min initial denaturation at 95 °C, followed by 40 cycles at 95 °C for 1 min each, 60 °C for 1 min and 72 °C for 2 min in a thermal cycler (7900HT, Thermo Fisher Scientific). The changes in mRNA levels were calculated by the delta-delta CT method using *β-actin* as an endogenous control.

### Transfection

LV12 cells were transfected with 50 nM of siRNA targeting *Amigo2* (SASI_Mm01_00045512), *Itgbl1* (SASI_Mm01_00116850), *Wisp1* (SASI_Mm01_00061775), or with a negative control siRNA (SIC-001, Sigma-Aldrich), using the Lipofectamine 2000 reagent (12566014, Thermo Fisher Scientific), for 24 h.

The pmirGLO vector (E1330, Promega, Madison, WI, USA) encoding *firefly luciferase* was transfected into LV12 and QRsP-11 cells. Stable clones were selected in the presence of 300 μg/ml neomycin (G418, Cellgro, Herndon, VA, USA). The clones were designated LV12-Luc cells and QRsP-11-Luc cells.

The pEZ-M02-*Amigo2* expression vector (EX-Mm13004-M02) was purchased from GeneCopoeia (Rockville, MD, USA). An empty vector was generated by removing the *Amigo2* insert using restriction digestion as follows. The pEZ-M02-*Amigo2* plasmid was digested with EcoRI and NotI (R0101S and R0189L, respectively, New England Biolabs, Beverly, MA, USA). 5′ overhangs were filled by using KOD DNA polymerase (KOD-101, Toyobo, Osaka, Japan) to generate blunt ends, which were ligated together using Ligation high (LGK-101, Toyobo). QRsP-11 cells were transfected with the *Amigo2* expression vector (QRsP-11-Amigo2) or the empty vector (QRsP-11-V) and stable clones were selected with G418 as described above.

### Western blot analysis

Protein was extracted from cells in ice-cold lysis buffer (1% NP40, 50 mM Tris (pH 7.5), 165 mM NaCl, 10 mM EGTA, 1 mM Na_3_VO_4_, 10 mM NaF, 1 mM PMSF, 10 μg/ml aprotinin, and 10 μg/ml leupeptin). One hundred micrograms of protein was subjected to 10% SDS-PAGE under a reducing condition and then blotted to a polyvinylidene fluoride membrane (ISEQ00010, Merck Millipore, Darmstadt, Germany). This membrane was incubated with the mouse monoclonal anti-Amigo2 antibody diluted 1:25 (clone G-7; sc-373699, Santa Cruz Biotechnology, Santa Cruz, CA, USA) or with the mouse monoclonal anti-β-actin antibody diluted 1:2000 (clone AC-15; A5441, Sigma Aldrich), and then with peroxidase-conjugated goat polyclonal anti-mouse IgG antibody diluted 1:2000 (PM009-7; Medical & Biological Laboratories, Nagoya, Japan). The signals were detected using an enhanced chemiluminescence ECL kit (RPN2232, GE Healthcare, Buckinghamshire, UK). The intensity was quantified by ImageJ 1.49 v.

### *Ex vivo* bioluminescent imaging for assessment of tumour cell attachment to liver

Four hours and/or 7 days after intrasplenic injection of 5 × 10^6^ LV12-Luc or QRsP-11-Luc cells, the livers were removed and transferred into D-luciferase solution (300 μg/ml; 126-05116, Wako). Photons from firefly luciferase were counted by using the IVIS imaging system (Xenogen, Alameda, CA, USA) according to the manufacturer’s instructions. Data were analysed using the Living Image software (Xenogen).

### Immunohistochemistry

All tissue samples were fixed with a 10% formalin solution. The samples were dehydrated, embedded in paraffin, and cut into 4 μm sections. Immunohistochemistry was performed using the streptavidin-biotin-peroxidase complex method or a Histofine mouse stain kit (414322 F, Nichirei, Tokyo, Japan). The sections were stained with antibodies against firefly luciferase (1:10000 dilution; AB3256-100UL, Chemicon, Temecula, CA, USA) or Amigo2 (1:50 dilution; sc-373699, Santa Cruz Biotechnology). Antigen retrieval was performed by proteinase K digestion (firefly luciferase) or autoclave treatment (Amigo2). Immunoreactions were visualised with diaminobenzidine and the sections were counterstained with hematoxylin. For assessment of the expression of Amigo2, five fields were chosen at random and examined at ×400 magnification. The staining intensity on the cell surfaces was scored as 0 (negative), 1 (weak), or 2 (moderate to strong).

### Clinical samples

The analysis of clinical materials was approved by the institutional review board of the ethics committee of Tottori University Hospital (Permission No. 1558), and written informed consent was received from each patient. All the experimental procedures were performed in accordance with guidelines of the ethics committee of Tottori University. The tissue samples were obtained from primary human colon and gastric carcinomas and their matched liver metastases.

### PrognoScan analysis

Correlations between the expression of *Amigo2* and prognosis in colon cancer patients were analysed using the PrognoScan database (http://www.abren.net/PrognoScan/)^52^ and the publicly available Gene Expression Omnibus (http://www.ncbi.nlm.nih.gov/geo) with the accession number GSE17536. The patients’ clinical and pathological characteristics including tumour stage have been reported elsewhere^53^.

### Statistical analyses

The significance of the differences in the metastatic incidences were calculated by the *X*^2^ test. Student’s *t*-test was used to evaluate the differences in liver/body weight ratio, survival period, the number of metastatic nodules/foci, adherence to endothelial cells, quantification of gene expression, total flux of livers and staining intensity.

## Additional Information

**How to cite this article:** Kanda, Y. *et al. Amigo2*-upregulation in Tumour Cells Facilitates Their Attachment to Liver Endothelial Cells Resulting in Liver Metastases. *Sci. Rep.* **7**, 43567; doi: 10.1038/srep43567 (2017).

**Publisher's note:** Springer Nature remains neutral with regard to jurisdictional claims in published maps and institutional affiliations.

## Supplementary Material

Supplementary Information
