# Supplementary material for: Amigo2-upregulation in Tumour Cells Facilitates Their Attachment to Liver Endothelial Cells Resulting in Liver Metastases
Source: Sci Rep. 2017 Mar 8;7:43567. doi: 10.1038/srep43567 (PMC5341090; doi:10.1038/srep43567)
Supplement: Supplementary Information [file srep43567-s1.pdf]

Supplementary Information

Amigo2-upregulation in Tumour Cells Facilitates Their Attachment to Liver Endothelial Cells Resulting in Liver Metastases

Yusuke Kanda[1], Mitsuhiko Osaki[1,2], Kunishige Onuma[1], Ayana Sonoda[1], Masanobu Kobayashi[3], Junichi Hamada[3], Garth L. Nicolson[4], Takahiro Ochiya[5] and Futoshi Okada[1,2,*]

[1]Division of Pathological Biochemistry, Tottori University Faculty of Medicine, Yonago, Japan
[2]Chromosome Engineering Research Center, Tottori University, Yonago, Japan
[3]Health Sciences University of Hokkaido, School of Nursing and Social Services, Ishikari-Tobetsu, Japan
[4]Department of Molecular Pathology, The Institute for Molecular Medicine, South Laguna Beach, CA, USA
[5]Division of Molecular and Cellular Medicine, National Cancer Center Research Institute, National Cancer Center Hospital, Tsukiji, Chuo-ku, Tokyo, Japan

[*]Address correspondence and reprint requests to:
Futoshi Okada, Ph.D.
Division of Pathological Biochemistry, Tottori University Faculty of Medicine, 86 Nishicho, Yonago 683-8503, Japan
Phone: +81-859-38-6241; Fax: +81-859-38-6240;
E-mail: fuokada@med.tottori-u.ac.jp

Supplementary Figure S1.

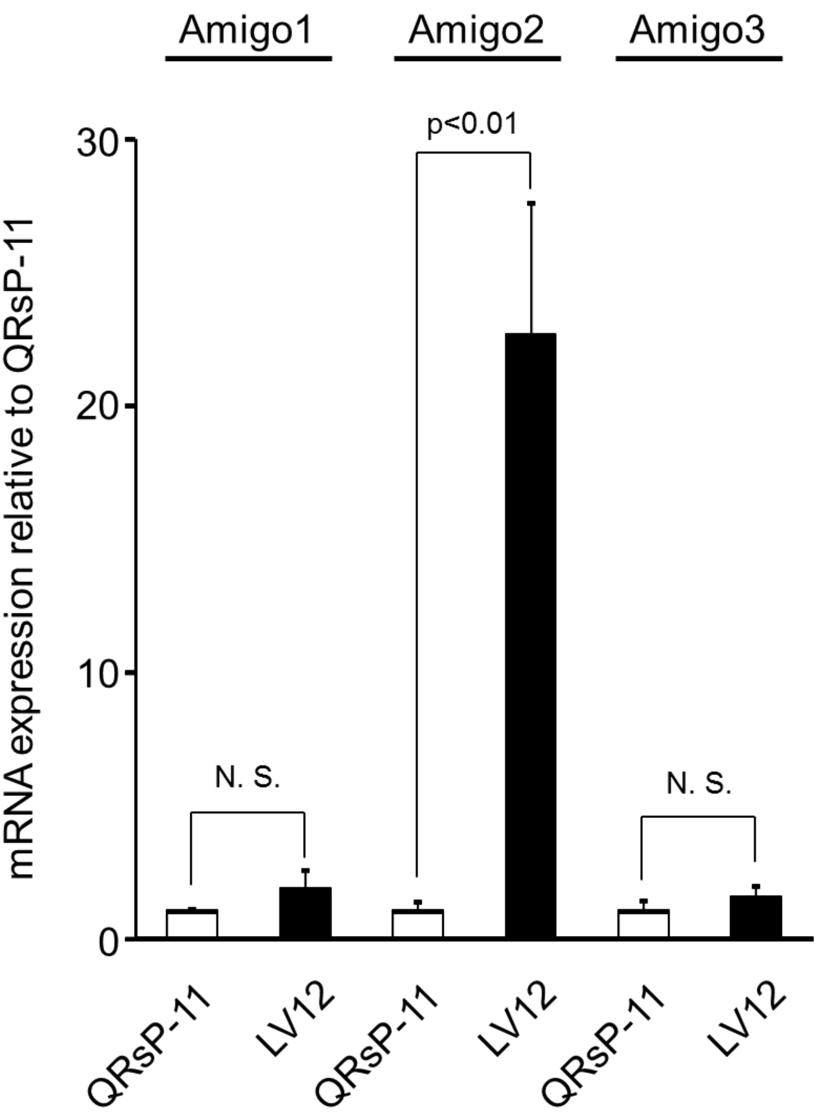

Assessment of Amigo family mRNA expression in LV12 and QRsP-11 cells

The mRNA expression levels of *Amigo1*, *Amigo2* and *Amigo3* in LV12 and QRsP-11 cells were evaluated using qRT-PCR. Bar graphs show means ± SD (n = 4 in each group)

Supplementary Figure S2.

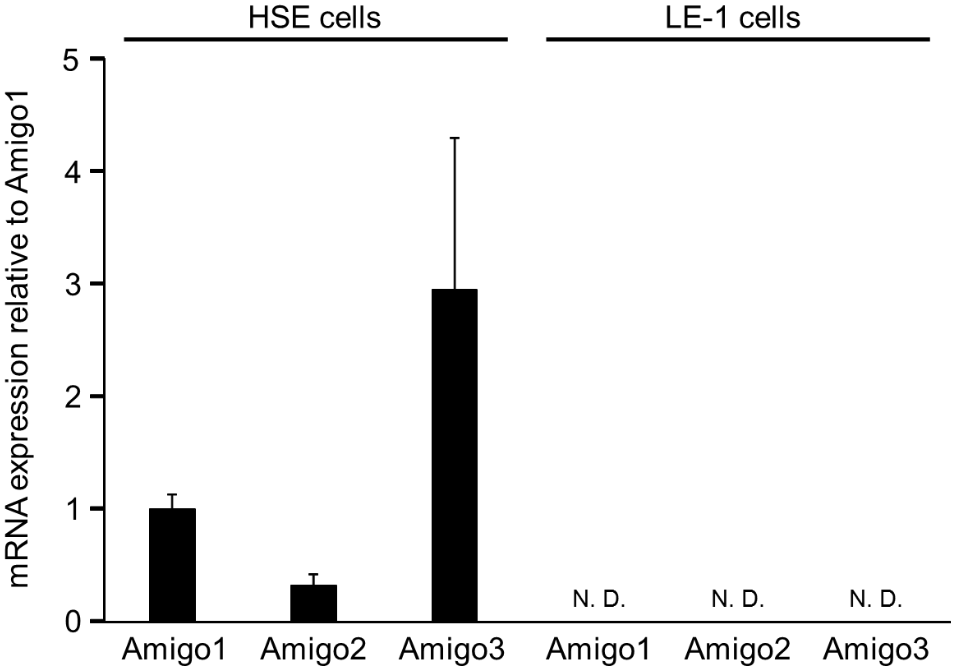

mRNA expression profiles of Amigo family members in HSE and LE-1 cells

Levels of *Amigo1*, *Amigo2* and *Amigo3* mRNAs in HSE and LE-1 cells were measured using qRT-PCR. Bar graphs show means ± SD (n = 4 in each group).

Supplementary Figure S3.

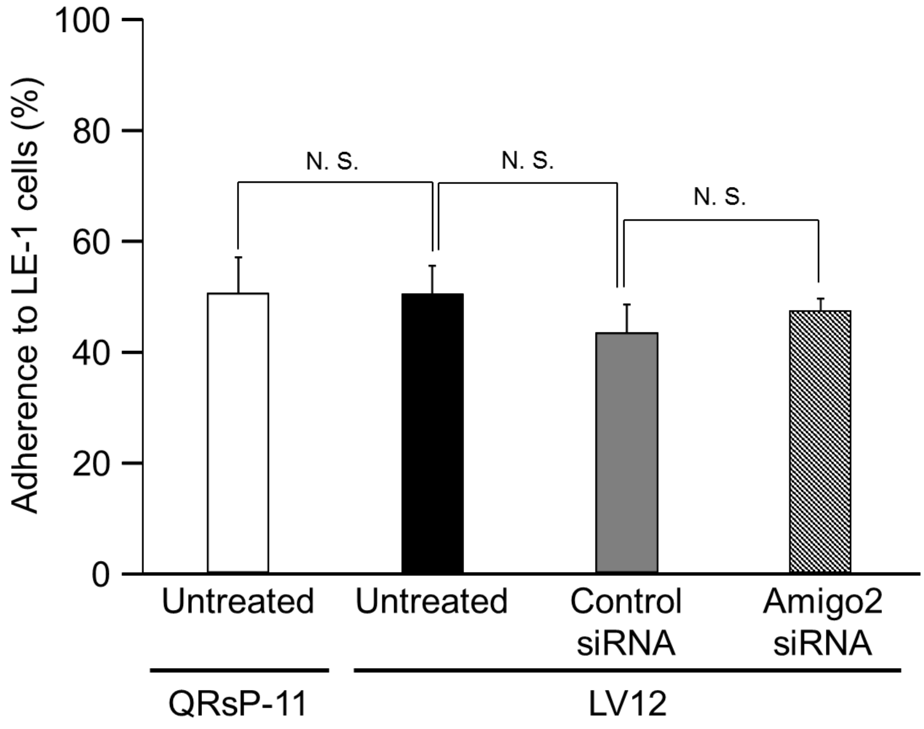

Amigo2 was not required for LV12 cell adhesion to LE-1 lung endothelial cells

LV12 cells were transfected with *Amigo2* or control siRNA and their adhesion to LE-1 lung endothelial cells was then assayed. Bar graphs show means ± SD (n = 5 in each group).

Supplementary Figure S4.

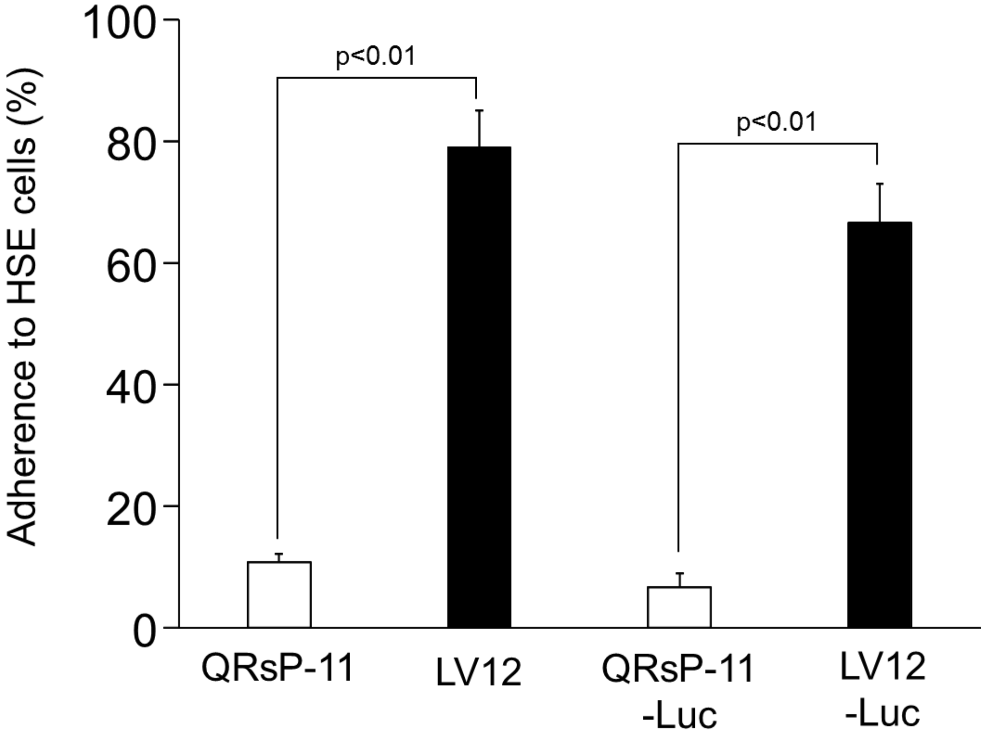

Adhesion properties of tumour cells to HSE liver endothelial cells were not changed by introduction of a luciferase expression vector

The adhesion properties of LV12-Luc or QRsP-11-Luc cells to HSE liver endothelial cells were assessed using an *in vitro* adhesion assay. Bar graphs show means ± SD (n = 10 in each group).

Supplementary Figure S5.

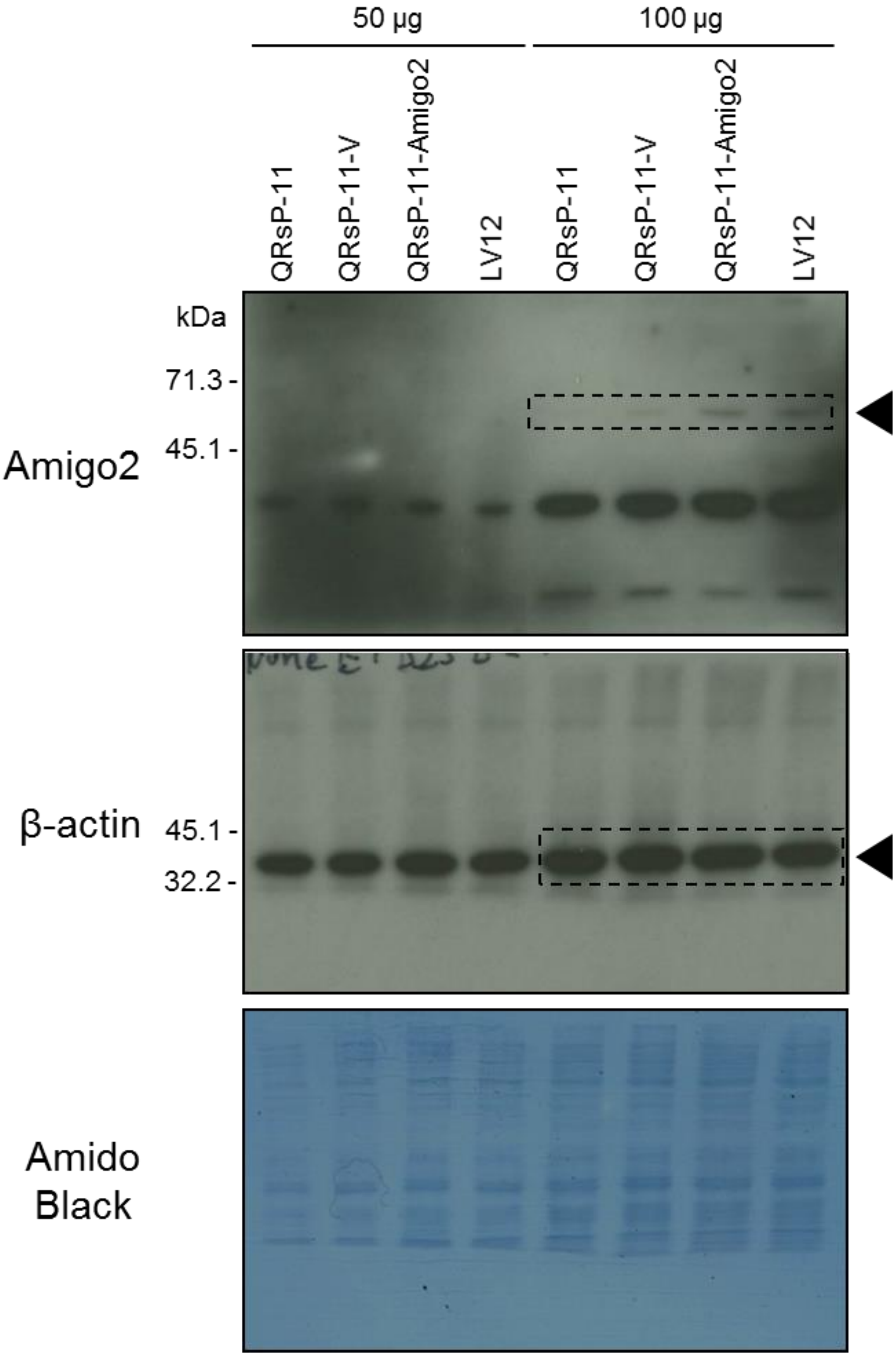

Whole western blots from Figure 5a in the manuscript

The top panel is the whole gel image for western blot using anti-Amigo2 antibody in Figure 5a. The middle panel is the whole gel image for western blot using anti-β-

actin antibody in Figure 5a. The bottom panel is 0.1% Amido Black 10B (015-02192, Wako, Osaka, Japan) staining for confirmation of equivalence of the loading protein in each lane. The mouse monoclonal anti-Amigo2 antibody is described in the manufacturer's website (https://www.scbt.com/scbt/product/amigo2-antibody-g-7?productCanUrl=amigo2-antibody-g-7&_requestid=916644) and datasheet (https://datasheets.scbt.com/sc-373699.pdf).

Supplementary Table S1.

Changes in mean survival in days, liver-to-body weight ratios and Amigo2 expression following in vivo selection of a subline with high liver-metastatic properties

Cell line[a]	No. of mice examined	Mean survival in days	Liver-to-body weight ratios (%)	Relative Amigo2 mRNA expression[b]
QRsP-11	5	17.0 ± 4.0	8.4 ± 4.8	1.0 ± 0.2
LV1	5	18.4 ± 3.4	6.5 ± 3.4	3.0 ± 0.4[d]
LV2	3	16.0 ± 0.0	20.6 ± 16.4	3.1 ± 0.2[d]
LV3	4	12.8 ± 2.9	6.9 ± 1.2	1.7 ± 0.4
LV4	3	21.0 ± 2.6	7.0 ± 2.2	9.4 ± 0.6[d]
LV5	3	20.3 ± 2.3	20.6 ± 19.0	18.0 ± 1.1[d]
LV6	5	21.4 ± 5.2	10.9 ± 8.9	15.7 ± 1.0[d]
LV7	4	20.3 ± 0.5	22.8 ± 20.6	30.6 ± 3.2[d]
LV8	3	20.3 ± 3.2	32.4 ± 2.3[d]	17.1 ± 0.7[d]
LV9	5	18.4 ± 1.3	14.7 ± 13.0	17.2 ± 0.8[d]
LV10	3	18.7 ± 1.2	30.7 ± 21.9	15.3 ± 0.8[d]
LV11	3	18.7 ± 2.3	28.4 ± 11.4[c]	21.1 ± 2.1[d]
LV12	7	13.3 ± 1.6[c]	28.2 ± 9.6[d]	19.1 ± 0.3[d]

a. QRsP-11 cells (1×10^6) were injected into mouse spleens. The mice were sacrificed when they appeared moribund. Tumour cells from liver metastases were cultured and used for sequential selection cycles.

b. *Amigo2* mRNA levels are indicated as fold expression relative to QRsP-11 cells.

c. $p<0.05$ *versus* QRsP-11 cells.

d. $p<0.01$ *versus* QRsP-11 cells.

Supplementary Table S2.

Classification of cell adhesion-related molecules upregulated or downregulated in LV12 cells compared with QRsP-11 cells

Upregulated gene		Downregulated gene	
Name	Fold change[a]	Name	Fold change[a]
**Integrin-related gene**			
Integrin beta-like 1 (Itgbl1)	16.5	Integrin, alpha E, epithelial-associated (Itgae)	0.3
Integrin beta 7 (Itgb7)	4.3		
**Immunoglobulin-related gene**			
Immunoglobulin superfamily, member 4A (Igsf4a)	3.6	Activated leukocyte cell adhesion molecule (Alcam)	0.1
Testicular cell adhesion molecule 1 (Tcam1)	2.9	Biregional cell adhesion molecule-related/down-regulated by oncogenes (Cdon) binding protein (Boc)	0.4
Poliovirus receptor (Pvr)	2.7		
CD4 antigen (Cd4)	2.3		
		F11 receptor (F11r)	0.4
		Coxsackievirus and adenovirus receptor (Cxadr)	0.4
		Neural cell adhesion molecule 1 (Ncam1)	0.5
		Neighbor of Punc E11 (Nope)	0.5
		CD47 antigen (Cd47)	0.5
**Cadherin-related gene**			
Protocadherin 9 (Pcdh9)	4.4	Protocadherin beta 14 (Pcdhb14)	0.2
		FAT tumor suppressor homolog 1 (Drosophila) (Fat1)	0.2
		Protocadherin beta 20 (Pcdhb20)	0.2
		Cadherin-like 26 (Cdh26)	0.4
		Protocadherin beta 17	0.4

		(Pcdhb17)	
		Protocadherin beta 22 (Pcdhb22)	0.5
**Others**			
Amphoterin-induced gene and open reading frame 2 (Amigo2)	15.1	Procollagen, type VI, alpha 1 (Col6a1)	0.1
Wnt-1-inducible signalling pathway protein 1 (Wisp1)	7.4	Palladin, cytoskeletal associated protein (Palld)	0.1
Nexilin (Nexn)	5.0	Endothelial differentiation sphingolipid G-protein-coupled receptor 1 (Edg1)	0.1
Contactin associated protein-like 4 (Cntnap4)	3.3	Neuropilin 1 (Nrp1)	0.2
Unknown	3.0	Spondin 2, extracellular matrix protein (Spon2)	0.2
Procollagen, type VI, alpha 3 (Col6a3)	2.9	Unknown	0.2
Pinin (Pnn)	2.8	Fibronectin 1 (Fn1)	0.2
Killer cell lectin-like receptor, subfamily A, member 18 (Klra18)	2.7	Plakophilin 2 (Pkp2)	0.2
Phosphatidylinositol 3-kinase, regulatory subunit, polypeptide 1 (p85 alpha) (Pik3r1)	2.4	Procollagen, type VI, alpha 2 (Col6a2)	0.3
Transglutaminase 2, C polypeptide (Tgm2)	2.4	Fibronectin leucine rich transmembrane protein 2 (Flrt2)	0.3
Thrombospondin 4 (Thbs4)	2.3	Rho family GTPase 3 (Rnd3)	0.3
Claudin 3 (Cldn3)	2.2	B-cell leukemia/lymphoma 6 (Bcl6)	0.3
Killer cell lectin-like receptor subfamily A, member 14 (Klra14)	2.2	Beta-1,4-N-acetyl-galactosaminyl transferase 2 (B4galnt2)	0.3
G protein-coupled receptor 98 (Gpr98)	2.1	Chemokine (C-X3-C motif) ligand 1 (Cx3cl1)	0.3
Connective tissue growth factor (Ctgf)	2.0	Embryonal Fyn-associated substrate (Efs)	0.4
Kit ligand (Kitl)	2.0	Elastin microfibril interfacer 2 (Emilin2)	0.4
		von Willebrand factor A domain containing 1 (Vwa1)	0.4

	Procollagen, type V, alpha 2 (Col5a2)	0.5
	LIM domain containing preferred translocation partner in lipoma (Lpp)	0.5
	Scavenger receptor class F, member 2 (Scarf2)	0.5
	Nidogen 1 (Nid1)	0.5
	Procollagen, type V, alpha 1 (Col5a1)	0.5
	Unknown	0.5
	Thrombospondin 1 (Thbs1)	0.5

a. Cell adhesion-related genes whose expression levels had changed more than 2-fold or less than 0.5-fold in LV12 cells compared with QRsP-11 cells are indicated.

Supplementary Table S3.

Primer sequences used in this study

Target	Strand	Sequence
Amigo1	Forward	5′-GAATGCCTTTGAGGACATGG-3′
	Reverse	5′-CTTCAGCTTGTTGGAGGACAG-3′
Amigo2	Forward	5′-GAGGCGACCATAATGTCGTT-3′
	Reverse	5′-CGGGCACCTTAGATAGGTTTT-3′
Amigo3	Forward	5′-CAGCCTCTCAGGATGGTAGC-3′
	Reverse	5′-CAGGGTGGTAAAGCCTGTGT-3′
Itgbl1	Forward	5′-AGCAACCAGATGTGCAAGAA-3′
	Reverse	5′-CCATGGCCTCCACATACTTC-3′
Wisp1	Forward	5′-TTCTGCAAGTGGCCATAGGT-3′
	Reverse	5′-ACTGGCCAGGGACTCTCAC-3′
β-actin	Forward	5′-AGAGGGAAATCGTGCGTGAC-3′
	Reverse	5′-CAATAGTGATGACCTGGCCGT-3′
